# Novel Implementation Strategy to Electronically Screen and Signpost Patients to Health Behavior Apps: Mixed Methods Implementation Study (OptiMine Study)

**DOI:** 10.2196/34271

**Published:** 2022-07-11

**Authors:** Zarnie Khadjesari, Tracey J Brown, Alex T Ramsey, Henry Goodfellow, Sherine El-Toukhy, Lorien C Abroms, Helena Jopling, Arden Dierker Viik, Michael S Amato

**Affiliations:** 1 Behavioural and Implementation Science Research Group School of Health Sciences University of East Anglia Norwich United Kingdom; 2 Department of Psychiatry Washington University School of Medicine St Louis, MO United States; 3 Department of Primary Care and Population Health University College London London United Kingdom; 4 Division of Intramural Research The National Institute on Minority Health and Health Disparities The National Institutes of Health Bethesda, MD United States; 5 Department of Prevention and Community Health Milken Institute School of Public Health The George Washington University Washington DC, DC United States; 6 Department of Public Health West Suffolk NHS Foundation Trust Bury St Edmunds United Kingdom; 7 Truth Initiative Washington DC, DC United States

**Keywords:** electronic health record, EHR, alcohol reduction, electronic messages, proactive messages, proactive outreach, smoking cessation, tobacco use, alcohol use, alcohol, smoking, mobile health, mHealth, mobile app

## Abstract

**Background:**

Behavior change apps have the potential to provide individual support on a population scale at low cost, but they face numerous barriers to implementation. Electronic health records (EHRs) in acute care hospitals provide a valuable resource for identifying patients at risk, who may benefit from behavior change apps. A novel, emerging implementation strategy is to use digital technologies not only for providing support to help-seeking individuals but also for signposting patients at risk to support services (also called *proactive referral* in the United States).

**Objective:**

The OptiMine study aimed to increase the reach of behavior change apps by implementing electronic signposting for smoking cessation and alcohol reduction in a large, at-risk population that was identified through an acute care hospital EHR.

**Methods:**

This 3-phase, mixed methods implementation study assessed the acceptability, feasibility, and reach of electronic signposting to behavior change apps by using a hospital’s EHR system to identify patients who are at risk. Phase 1 explored the acceptability of the implementation strategy among the patients and staff through focus groups. Phase 2 investigated the feasibility of using the hospital EHR to identify patients with target risk behaviors and contact them via SMS text message, email, or patient portal. Phase 3 assessed the impact of SMS text messages sent to patients who were identified as smokers or risky drinkers, which signposted them to behavior change apps. The primary outcome was the proportion of participants who clicked on the embedded link in the SMS text message to access information about the apps. The acceptability of the SMS text messages among the patients who had received them was also explored in a web-based survey.

**Results:**

Our electronic signposting strategy—using SMS text messages to promote health behavior change apps to patients at risk—was found to be acceptable and feasible and had good reach. The hospital sent 1526 SMS text messages, signposting patients to either the National Health Service Smokefree or Drink Free Days apps. A total of 13.56% (207/1526) of the patients clicked on the embedded link to the apps, which exceeded our 5% a priori success criterion. Patients and staff contributed to the SMS text message content and delivery approach, which were perceived as acceptable before and after the delivery of the SMS text messages. The feasibility of the SMS text message format was determined and the target population was identified by mining the EHR.

**Conclusions:**

The OptiMine study demonstrated the proof of concept for this novel implementation strategy, which used SMS text messages to signpost at-risk individuals to behavior change apps at scale. The level of reach exceeded our a priori success criterion in a non–help-seeking population of patients receiving unsolicited SMS text messages, disconnected from hospital visits.

**International Registered Report Identifier (IRRID):**

RR2-10.2196/23669

## Introduction

More than one-third of cancers are preventable by adopting a healthier lifestyle, such as stopping smoking and reducing risky drinking; tackling these health behaviors would lead to significant health benefits, minimize comorbidity, and reduce the burden on the National Health Service (NHS) [1-3]. The traditional approach to health promotion in the hospital environment is centered on health professionals providing verbal advice or information in leaflets, with or without the offer of referral to a lifestyle service. This approach relies on the clinician’s knowledge, skills, and confidence; time; and the availability of health promotion literature, of which all are significant barriers to delivery [4,5]. Attempts have been made to boost health promotion activities in acute care hospitals in England. For example, (1) financial incentives have been provided for hospitals that undertake screening and brief advice for smoking and risky drinking (2018-2020) [6]; (2) brief advice on health behaviors through opportunistic day-to-day interactions between patients and health care professionals is promoted via Making Every Contact Count, an NHS initiative [7]; and (3) public health specialists are increasingly being embedded into the acute care context in recognition of the links between health behaviors and chronic illnesses, such as cancer, and the pressure this puts on the NHS. However, these interventions are typically resource-intensive or have been discontinued owing to resource constraints.

Digital interventions, such as behavior change websites or apps, have been developed to bridge the evidence-to-practice gap. They can be used as an alternative or adjunct to face-to-face delivery and have a substantial evidence base as interventions [8-12]. Digital interventions can provide effective individual support on a population scale at low cost [13]. They overcome barriers to delivering face-to-face interventions, such as removing the stigma associated with seeking help and alleviating pressure on busy health professionals to deliver brief interventions. Digital interventions for addictive behaviors are more commonly offered in higher education, primary care, and community settings (although far from routine) but rarely in the context of acute health services [8,9,14-16]. Furthermore, the implementation of effective digital interventions is often not considered and relies on the help-seeking behavior of motivated individuals. Another underused digital resource in the acute care setting is the electronic health record (EHR) for screening patients at risk. Although screening is commonplace for promoting medication adherence, vaccine uptake, or cancer screening, systematic screening for health behaviors (such as risky alcohol consumption and tobacco smoking) for signposting to support services is in its infancy.

We developed a novel implementation strategy that uses the combined potential of three existing technologies to bridge the evidence-to-practice gap: (1) acute care hospital EHR for identifying at-risk individuals at scale, (2) health promotion apps and websites that provide behavior change support at an individual level, and (3) SMS text messages that are commonly used by health services to communicate with patients. Using electronic messages (such as SMS text message or email) to signpost (referred to as *proactive outreach* in the United States [17-19]) patients who are at risk, identified by the EHR, to health promotion apps is cheap, efficient, quick to implement using existing infrastructure, and scalable to other health behaviors and health care settings and has the potential to achieve behavior change at the population level. Although SMS text messaging has been used successfully as a treatment tool for smoking cessation [11], using it as a primary channel for outreach and enrollment to support services or treatment represents a novel application with preliminary success. Krebs et al [18] used outreach SMS text messages in a large New York health system to connect patients to quitline counseling. Furthermore, Abroms et al [20] used SMS text messaging to connect patients in the emergency department to a smoking cessation SMS text messaging program or quitline counseling.

So far, no previous studies have evaluated the ability of electronic messages to signpost patients who are not seeking help to behavior change apps, disconnected from hospital visits. The aim of this 3-phase, mixed methods implementation study (the OptiMine study) was to explore the acceptability (phase 1), feasibility (phase 2), and reach (phase 3) of electronic messages to signpost patients who smoke tobacco and drink alcohol at risky levels to behavior change apps. The primary outcome of phase 1 was a qualitative synthesis of patient-perceived attributes of signposting. The primary outcome of phase 2 was the estimated size and characteristics of the population that may be reached by the intervention. Findings from these 2 phases informed the design of phase 3, the primary outcome of which was the proportion of contacted patients who followed the signpost to access more information about the promoted behavior change apps (ie, the *click rate*). The approach and methods have been reported in our published protocol [21].

## Methods

### Ethics Approval

The NHS Research Ethics Committee and the Health Research Authority approved this study in June 2019. EHR data were accessed and analyzed by staff within the West Suffolk NHS Foundation Trust (WSFT) information service team. Patients at risk were identified by the WSFT information service team and received electronic messages directly from the hospital. All analyses were conducted by the WSFT information service and public health teams. The study team members had access only to anonymized, aggregated data. Authorization for using EHR data at the WSFT was provided by the Information Governance Team via Data Protection Impact Assessment (approval date: September 20, 2019) [22].

### Theoretical Frameworks

We used the taxonomy of implementation outcomes by Proctor et al [23] to design this implementation study, focusing on acceptability, feasibility, and reach. Acceptability and feasibility are important precursors for effective uptake and reach of an intervention and were explored using qualitative focus groups (acceptability before implementation), a web-based survey (acceptability after implementation), and routinely collected data from the EHR (feasibility). We assessed *reach* as our measure of implementation success, operationalized as the proportion of patients who engaged with our SMS text message by clicking on an embedded link to access free apps to support health behavior change. The topic guides and survey exploring the acceptability of delivering electronic messages to patients were informed by the Perceived Attributes of eHealth Innovations [24]—an extension of the Diffusion of Innovations Theory [25], which has been applied through a validated questionnaire to test the acceptability of a digital innovation [26]. Further information on the model and its application in this study is provided in our published protocol [21].

### Setting

The research was conducted between April 2019 and July 2020 within the West Suffolk Hospital, an acute NHS provider renowned for its world-leading delivery of care using digital technologies, that is, an acute Global Digital Exemplar Trust [27]. eCare (trade name: Cerner Millenium), the EHR system at the WSFT, was launched in 2016 and includes records for all outpatients and inpatients registered with the hospital. The EHR contains contact information, demographics, and health data such as chronic disease status needed for this study. Data on smoking and alcohol consumption status were collected as part of the lifestyle screening survey or the Activities of Daily Living assessment, routinely provided to patients on admission to the hospital. The hospital is situated in the rural region of West Suffolk in the East of England, where population characteristics are similar to those of the general population in England, with slightly higher proportions of individuals aged >65 years and White British residents [28].

### Implementation Strategy and Behavior Change Apps

We electronically signposted patients to the NHS Smokefree and Drink Free Days apps. Public Health England has developed the Smokefree and the Drink Free Days apps as part of their One You campaign for supporting healthy lifestyles. These apps are freely available on the web [29] and are heavily promoted in the United Kingdom mass media. The apps are theoretically informed and use evidence-based behavior change techniques, such as goal setting and self-monitoring. This mixed methods implementation study explored acceptability (phase 1) and feasibility (phase 2) as important precursors for the reach of our implementation strategy (phase 3).

### Phase 1: Acceptability of Electronic Signposting (Before Implementation)

Acceptability of the implementation strategy was explored before its delivery via focus groups. Patients and staff were invited to participate in face-to-face focus groups to explore their perspectives. Patients were eligible if they were smoking or drinking alcohol regularly. Eligible staff were those (1) in senior IT management roles, (2) responsible for administering lifestyle screening, or (3) involved in EHR data management and hospital communications. Patients were identified via volunteer coordinators, a news story on the hospital website, and a recruitment stall at the main hospital entrance. Staff were recruited directly through email invitation and a weekly staff newsletter. All participants were provided with a participant information sheet and a consent form. Patient focus groups were conducted on-site at the education center at WSFT, whereas staff focus groups were conducted in meeting rooms. Focus groups were audio-recorded and transcribed verbatim by a professional transcription company, removing any identifiable data. Transcripts were coded using NVivo (QSR International). Framework analysis was used to synthesize the findings of the focus groups, based on 3 domains of the Perceived Attributes Theory: compatibility (ie, degree to which electronic messaging was consistent with patient preferences), complexity (ie, degree to which electronic messaging was difficult to understand or act on), and relative advantage (ie, degree to which electronic messaging was superior to alternative or more traditional approaches) [24]. Subthemes were focused on the pragmatic development, refinement, and delivery of the implementation strategy.

### Phase 2: Feasibility of Electronic Signposting

Feasibility of using the EHR to identify patients at risk who have mobile phone numbers, email addresses, and patient portal access was explored through data mining. Patients with alcohol consumption or smoking status that had been recorded or updated within the past 13 months were included. The Activities of Daily Living and customized lifestyle screening assessments are used by the WSFT to record alcohol consumption and smoking status on admission to the hospital. The following data were extracted and aggregated from the EHR by a hospital-based information analyst: smoking status (yes or no), alcohol consumption status: Alcohol Use Disorders Identification Test–Consumption (AUDIT-C) score [30] (low risk: 0-4, at risk: 5-9, and dependent: 10-12), sex (woman or man), mobile phone number (yes or no), email address (yes or no), and patient portal access (yes or no). Frequencies and percentages of patients with valid data in each of the fields mentioned previously were reported to the study team, who had no direct access to the underlying data.

### Phase 3: Reach of Electronic Signposting

#### Participants

Eligible patients were adults (aged ≥18 years) recorded as smoking or drinking alcohol at risky levels within the past 13 months, with a valid mobile number. Patients were excluded if they were pregnant, were registered on the end-of-life pathway, or had opted out of communications from the hospital. As specified in the protocol, a minimum sample size of 383 per risk profile group would allow calculation of 95% CIs within a margin of –5% to +5% points, assuming a population proportion of 50% and a population size of 100,000 [21].

#### Procedure

**Figure 1:**
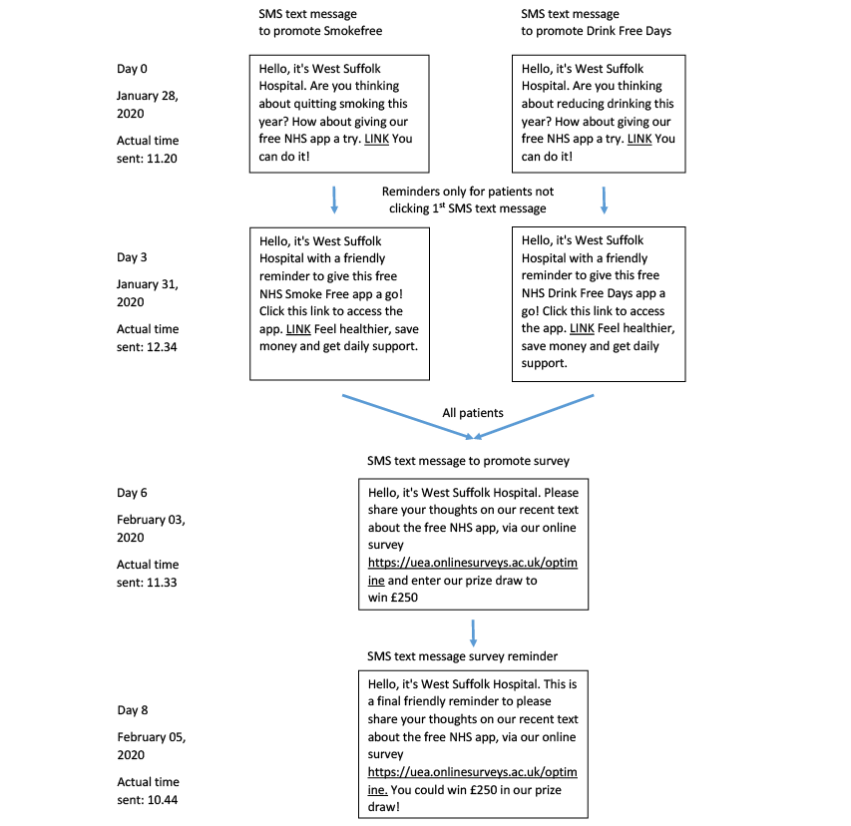
Flowchart of SMS text message content and delivery.

SMS text messages were selected as the most acceptable and feasible electronic format based on the findings of phase 1 and phase 2. Although the protocol sought to compare 3 risk profiles (exclusive smokers, exclusive risky drinkers, and both), to reach the a priori specified minimum sample size, it was necessary to collapse to two risk profiles: (1) exclusive smokers and (2) risky drinkers regardless of smoking status. The hospital’s SMS text messaging system was used to send an initial message to all the participants, signposting to either the Smokefree or Drink Free Days apps based on risk profile, with a second reminder message sent 3 days later to any participant who had not clicked the link yet. Unique link URLs were used to identify the participants who clicked the link. Figure 1 illustrates the content and delivery schedule of the messages.

#### Data Collection

To determine the characteristics of patients to whom SMS text messages were sent, data were retrieved from eCare, and analyses were performed by a public health manager with the support of coauthors MSA and ZK in July 2020. The following variables were extracted from the system and categorized as follows:

1. Name (for data linkage purposes only)
2. Hospital and NHS number (for data linkage purposes only)
3. Date of birth (for data linkage purposes only)
4. Age—categorized as 18-25, 26-35, 36-45, 46-55, 56-65, and >66 years
5. Sex—woman or man
6. Ethnicity—categories were merged to increase patient numbers in less-populated groups: any White background, any other background, and not known or not stated; refer to the study protocol for individual categories [21]
7. Postcode—used to calculate index of multiple deprivation decile—grouped into quintiles
8. Date of smoking or alcohol consumption screening at the hospital—categorized as days since screening: 1-4, 4-7, 7-10, 10-13, and >13 months
9. SMS text message sent, targeting smoking cessation or alcohol reduction
10. Embedded link to apps clicked or not clicked

#### Indices of Multiple Deprivation

Postcodes were entered into the government multiple deprivation lookup to obtain the deciles of multiple deprivation for each patient [31], where decile 1 represents the most deprived 10% of the population and decile 10 represents the least deprived 10% of the population.

#### Recency of Screening

The number of days since patients were screened for smoking or alcohol consumption was calculated by subtracting the date of smoking or alcohol screening from the date on which the smoking or alcohol SMS text message was sent. This was rounded to a whole number.

#### Health Data

The long-term health conditions stored as structured data in eCare were audited against the whole medical record to determine its suitability for use. Auditors used inpatient, emergency department, and general practice notes to collate patients’ medical history. More than half of the manually audited records found different health data than those retrieved from the patient records. Thus, the health data were considered as too incomplete and inaccurate to be included in the study. Refer to the study protocol for the list of long-term health conditions originally intended for extraction from eCare [21].

#### Missing Data in AUDIT-C Fields

A large proportion of the records (1424/1975, 72.1%) that documented alcohol status had missing data in the AUDIT-C fields. Scores were calculated from the available fields, imputing a value of zero for missing fields, based on information from hospital staff that fields were most likely skipped because they were not applicable (eg, erroneously left blank instead of selecting *0*). Patients with missing data and calculated AUDIT-C scores of 7 to 8 were excluded from the study because it was not possible to be confident that these patients were not dependent drinkers, for whom the intervention would be clinically inappropriate.

#### Statistical Modeling

We determined a priori that 5% reach, as evidenced by clicking on the embedded links to the apps, would constitute a clinically meaningful level of reach, given the low-burden and scalable nature of the interventions [21]. This success criterion was based on the rationale that reaching even this modest proportion of non–help-seeking individuals with high-risk drinking and smoking behaviors via unsolicited SMS text messages at a time disassociated with their last hospital visit could have great impact at a population health level. The number of patients who clicked the embedded link within their respective SMS text message was reported along with their baseline characteristics. The denominator for reach rate was the total number of patients with that characteristic who received an SMS text message. R software (R Foundation for Statistical Computing) was used to model the logistic regression, where we used the glm function with a Poisson distribution. To investigate potential relationships of common demographic covariates and assess any effect of screening recency, the model included all patient-level variables that were available: sex, age group, ethnicity group, index of multiple deprivation quintile, and days since screening group. Relative risks (RRs) with 95% CIs for each characteristic were derived from the model in R, using exponentiated values of the model coefficients.

### Acceptability of Electronic Signposting (After Implementation)

Acceptability of the implementation strategy was also explored after its delivery via a web-based survey. Eligible patients were those to whom a signpost SMS text message was sent. Then, 6 days after the initial signpost SMS text message was sent, 2 additional SMS text messages were sent to the participants (an initial message and a reminder), inviting them to participate in a web-based survey about their views on receiving the signpost SMS text messages. Participant information sheets and consent forms were incorporated into the web-based survey. We used JISC web-based surveys, a free web-based platform designed for academic research and public sector organizations. Participants were given 15 days to complete the survey.

## Results

### Phase 1: Acceptability of Electronic Signposting (Before Implementation)

A total of 10 patients participated in 2 focus groups (group 1: n=3, 30% of the participants; group 2: n=6, 60% of the participants) and 1 individual interview (n=1, 10% of the participants; owing to low turnout for the focus group). A total of 14 staff members participated in 3 focus groups (group 1: n=5, 36% senior managers; group 2: n=1, 7% nurse and n=1, 7% pharmacy technician; group 3: n=7, 50% members of IT staff and communications officers). Patients’ ages were collected as categories and ranged from 46 to >66 years; 60% (6/10) were women. Most patients (7/10, 70%) were members of the hospital’s volunteer group and patient portal user group, with 20% (2/10) of them being members of the public and 10% (1/10) being members of staff identified via the recruitment stall. Findings from this phase suggested that most patients found SMS text messaging as the most acceptable form of electronic message for receiving communications. A more detailed summary of the key findings under each theme and subtheme is presented in Multimedia Appendix 1 (qualitative focus group findings), along with illustrative quotes and the approach that informed the development and delivery of electronic messages.

### Phase 2: Feasibility of Electronic Signposting

The dynamic nature of an acute care hospital EHR database was found to be an implementation challenge. The mining of data from eCare needed to be performed on different days, owing to the size of the data queries, and with different search strategies depending on the fields required. Furthermore, the results changed depending on the date on which the queries were run (both the denominators and numerators). Queries that were intended to generate data sets that would include the whole EHR population could only be achieved by limiting the number of variables in the data set; large queries often took several hours to run and were at risk of timing out before they had completed.

The total number of adult patients in the eCare system on October 9, 2019, was 228,982, of which 1092 (0.48%) adults were recorded as smokers (smoking and drinking status data extracted on January 10, 2020). Among the 0.74% (1702/228,982) of the patients who were recorded as drinking alcohol, 23.62% (402/1702) patients were recorded as at-risk drinkers. An additional 0.55% (1249/228,982) and 0.02% (51/228,982) of the patients were reported as drinking at low risk or dependent levels, respectively. Most patients (226,784/228,982, 99.04%) had missing data in the EHR regarding their tobacco or alcohol use status. Smoking and drinking status are only recorded in a way that is retrievable when a patient has an inpatient admission. The proportion of the catchment population that is admitted each year is typically 13% [32]. The proportions of admitted patients who were screened for smoking and alcohol use in the financial year 2018 to 2019 were reported by WSFT information service staff to be 64% and 68%, respectively.

Of the 228,982 patients, 146,171 (63.84%) had mobile phone numbers, of which 707 (0.48%) were recorded as smokers and 271 (0.19%) were recorded as at-risk drinkers. Of the 228,982 patients, the total number of patients with email addresses was 32,375 (14.14%), of which 137 (0.42%) were recorded as smokers and 115 (0.36%) were recorded as at-risk drinkers. The database of patient portal users was independent of the EHR, and could not be linked to the EHR by the WSFT information service staff. The numbers of patient portal users recorded as smoking and drinking could not be determined. Therefore, the consideration of the patient portal as a form of message delivery was discontinued in this study. Combined with the findings from phase 1, these feasibility findings from phase 2 reinforced the decision to use SMS text messages as the channel for the electronic messages.

### Phase 3: Reach of Electronic Signposting

#### Baseline Characteristics and Risk Profile Groups

On the basis of the findings in phase 2, the participant sample was mined from eCare on January 10, 2020. The sampling frame used the most recent admissions to the hospital with validated data to identify patients whose records were most likely to be up to date and accurate. A 13-month time frame (October 1, 2018, to November 30, 2019) was used to obtain a sufficiently large population of patients to meet the minimum sample size required, which was balanced against recency of the data. A total of 6521 people admitted during the time frame were aged ≥18 years, not on the end-of-life pathway, and not pregnant and had a mobile phone number recorded and either their smoking or alcohol status recorded. Owing to the relatively small number of patients who could be identified as exclusively consuming alcohol at risky levels but who were nonsmokers, all participants with risky drinking were combined into a single risk profile group, regardless of their smoking status. The resulting 2 risk profile groups (smoking only and risky drinking with or without smoking) represent a deviation in analysis from the protocol, which aimed to create 3 risk profile groups (smoking only, risky drinking only, and smoking and risky drinking). This deviation was necessary to meet our a priori minimum sample size of 383 participants per group, which was also specified in the protocol. The decision to collapse the risky drinking groups was based exclusively on the baseline data, before the analyses of outcomes. Ultimately, of the 1526 individuals, the selected sample included 1103 (72.28%) individuals who were recorded as smokers only, 276 (18.09%) individuals who were recorded as risky drinkers only, and 147 (9.63%) individuals who were recorded as both smokers and risky drinkers. Table 1 shows the baseline characteristics of the participants.

**Table 1:** Baseline characteristics.

Characteristics		Smoker only^a^ (n=1103)	Risky drinker with or without also being a smoker^b^ (n=423)
**Sex, n (%)**			
	Women	553 (50.14)	129 (30.5)
	Men	550 (49.86)	294 (69.5)
	Not recorded	N/A^c^	N/A
**Age (years), mean (SD)**		47.78 (17.68)	56.43 (16.27)
	18-25, n (%)	124 (11.24)	17 (4)
	26-35, n (%)	200 (18.13)	29 (6.9)
	36-45, n (%)	192 (17.41)	60 (14.2)
	46-55, n (%)	205 (18.59)	90 (21.3)
	56-65, n (%)	176 (15.96)	98 (23.2)
	66-75, n (%)	125 (11.33)	78 (18.4)
	>75, n (%)	81 (7.34)	51 (12.1)
	Not recorded	N/A	N/A
**Ethnicity, n (%)**			
	White	1021 (92.57)	395 (93.4)
	Mixed	2 (0.18)	1 (0.2)
	Asian or Asian British	2 (0.18)	1 (0.2)
	Black or Black British	5 (0.45)	0 (0)
	Other ethnic groups	21 (1.90)	8 (1.9)
	Not recorded	52 (4.71)	18 (4.3)
**Index of multiple deprivation (quintile), n (%)**			
	1	77 (6.98)	17 (4)
	2	279 (25.29)	92 (21.7)
	3	371 (33.64)	147 (34.8)
	4	251 (22.76)	111 (26.2)
	5	121 (10.97)	49 (11.6)
	Not recorded	4 (0.36)	7 (1.7)
**Recency of screening data (months), n (%)**			
	0-1	N/A	N/A
	1-3	119 (10.79)	54 (12.8)
	3-6	249 (22.57)	127 (30)
	6-12	485 (43.97)	209 (49.4)
	>12	250 (22.67)	33 (7.8)
	Not recorded	N/A	N/A

^a^Group includes all participants recorded as smoking and not drinking alcohol at risky levels.

^b^Group includes all participants recorded as drinking alcohol at risky levels, regardless of smoking status, owing to sample size considerations. The risky drinker status is defined as an Alcohol Use Disorders Identification Test–Consumption score of 5 to 10 if 3 of its items were completed or a score of 5 to 6 if only 2 items were completed.

^c^N/A: not applicable.

#### Reach

**Figure 2:**
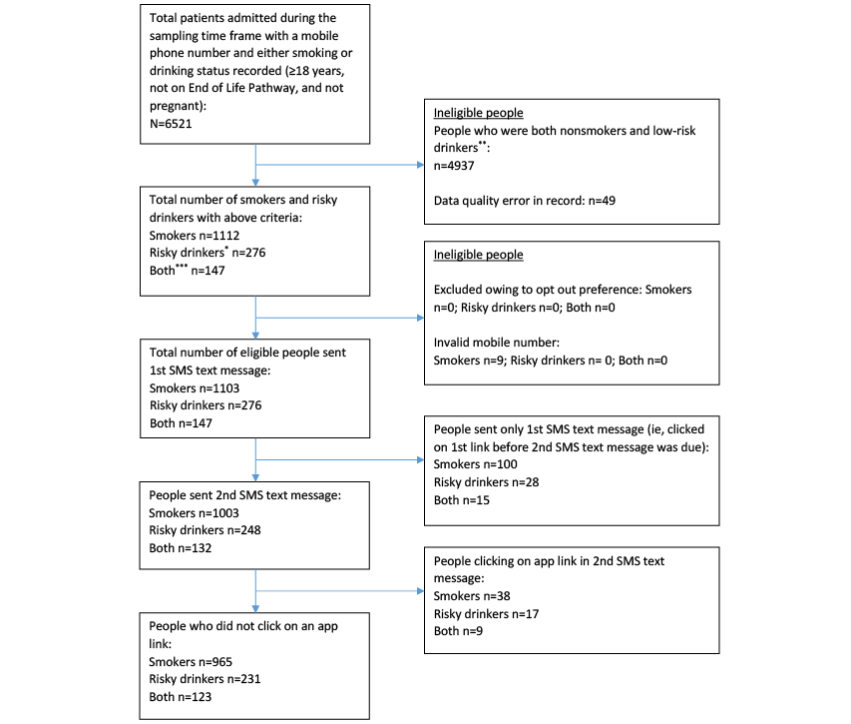
CONSORT (Consolidated Standards of Reporting Trials) diagram. *Alcohol Use Disorders Identification Test–Consumption (AUDIT-C) score of 5-10 if 3 fields populated or score of 5-6 if 2 fields populated; **AUDIT-C score<5; ***both=patients who are both smokers and risky drinkers.

In January 2020, an SMS text message was sent to 1526 patients, signposting them to either the NHS Smokefree (n=1103, 72.28%) or the Drink Free Days apps (n=423, 27.72%). A total of 13.56% (207/1526) of the participants clicked on the embedded link to the apps (smokers: 26/207, 12.56% and risky drinkers: 34/207, 16.43%), which exceeded our 5% a priori success criterion in both groups. Figure 2 shows a CONSORT (Consolidated Standards of Reporting Trials) diagram. Characteristics of patients who clicked the embedded link within the SMS text message versus the characteristics of those who did not click the embedded link are presented in Table 2. The only significant differences in baseline characteristics were observed among smokers. Among smokers, the lowest click rate was among participants aged ≥66 years (6.8%). Compared with this age group as reference, smoking participants aged 36 to 45 years (18.2%; RR=2.6; 95% CI 1.4-4.6) and those aged 56 to 65 years (13.1%; RR=1.98; 95% CI 1.05-3.73) were significantly more likely to click. Male smokers were significantly less likely to click than female smokers (10.2% vs 14.8%; RR=0.71; 95% CI 0.51-0.98). Although no significant differences by age were observed among risky drinkers, a numerically different distribution emerged, such that risky drinkers aged 56 to 65 years were the most likely to click the link (20.4%).

**Table 2:** Comparison of characteristics of patients who clicked versus those who did not click on links to apps within the SMS text message.

Characteristics			Smokers—patients who clicked versus those who did not click^a^ (n=1103)							Risky drinkers—patients who clicked versus those who did not click^b^ (n=423)				
			Patients who clicked, n (%)		RR^c^ (95% CI)		*P* value		Patients who clicked, n (%)			RR (95% CI)		*P* value
Overall click rate			138 (12.51)		—^d^		N/A^e^		69 (16.3)			N/A		N/A
**Sex**														
	Women	82 (14.83)		Reference		—		21 (16.3)			Reference		—	
	Men	56 (10.18)		0.71 (0.51-0.98)		.04		48 (16.3)			0.92 (0.56-1.51)		.74	
**Age (years)**														
	18-25	14 (11.29)		1.55 (0.76-3.13)		.23		1 (5.9)			0.38 (0.06-2.48)		.31	
	26-35	26 (13)		1.86 (1-3.44)		.05		1 (3.5)			0.22 (0.03-1.44)		.11	
	36-45	35 (18.23)		2.56 (1.42-4.64)		.002		10 (16.7)			1.18 (0.58-2.43)		.65	
	46-55	26 (12.68)		1.83 (0.99-3.39)		.06		17 (18.9)			1.24 (0.67-2.31)		.50	
	56-65	23 (13.07)		1.98 (1.05-3.73)		.03		20 (20.4)			1.34 (0.74-2.42)		.34	
	>66	14 (6.80)		Reference		—		20 (15.5)			Reference		—	
**Ethnicity**														
	White	120 (11.75)		Reference		—		65 (16.5)			Reference		—	
	People of color	6 (20)		1.63 (0.74-3.57)		.22		2 (20)			1.53 (0.39-6.06)		.54	
	Not known or not stated	12 (23.08)		2.12 (1.20-3.75)		.01		2 (11.1)			0.64 (0.17-2.44)		.52	
**Postcode (for index of multiple deprivation; quintile)**														
	1	10 (12.99)		0.96 (0.45-2.04)		.92		2 (11.7)			0.79 (0.18-3.47)		.76	
	2	37 (13.26)		0.96 (0.55-1.68)		.88		15 (16.3)			1.02 (0.45-2.30)		.96	
	3	49 (13.21)		0.96 (0.56-1.65)		.90		23 (15.7)			1.01 (0.48-2.16)		.97	
	4	26 (10.36)		0.76 (0.42-1.37)		.36		21 (18.9)			1.25 (0.58-2.69)		.57	
	5	16 (13.22)		Reference		—		8 (16.3)			Reference		—	
**Recency of screening data (months)**														
	1-4	28 (13.93)		1.11 (0.66-1.88)		.70		16 (16)			1.2 (0.17-8.33)		.85	
	4-7	29 (11.20)		0.88 (0.52-1.47)		.61		16 (12.8)			0.99 (0.14-6.91)		.99	
	7-10	34 (14.72)		1.18 (0.71-1.96)		.52		17 (15.5)			1.21 (0.17-8.44)		.85	
	10-13	24 (10.08)		0.80 (0.46-1.38)		.42		19 (23.2)			1.82 (0.26-12.72)		.54	
	>13	23 (13.22)		Reference		—		1 (16.7)			Reference		—	

^a^These messages were sent to patients recorded as smoking and not recorded as drinking alcohol at risky levels.

^b^These messages were sent to all patients recorded as drinking alcohol at risky levels, including those who were also recorded as smoking. These data were merged owing to the low number of patients recorded as drinking at risky levels.

^c^RR: relative risk.

^d^Not available.

^e^N/A: not applicable.

### Acceptability (After Implementation)

The survey was completed by 3.67% (56/1526) of participants. Among the 56 survey responders, 18 (32%) participants reported that they had clicked on the link within the SMS text message and 9 (16%) participants reported that they downloaded the app. Approximately two-thirds (36/56, 64%) of the participants found the messages to be at least slightly *helpful*. The message was found to be *not at all difficult* by almost all patients (55/56, 98%). Most participants (51/56, 91%) were happy with the wording of the SMS text message. Almost half of the participants (26/56, 46%) reported that they would like the hospital to deliver this service; however, an opt-out option was also popular (21/56, 38%). Most of the respondents (42/56, 75%) submitted free-text comments. Common positive themes were that the SMS text messages were supportive (7/56, 13%), easy to understand (6/56, 11%), and brief (6/56, 11%). Common negative themes were that the messages were irrelevant (9/56, 16%), not supportive (4/56, 7%), and unwanted or unexpected (3/56, 5%).

## Discussion

### Principal Findings

Our electronic signposting strategy that used SMS text messages to promote health behavior change apps to patients at risk via an acute care hospital EHR was found to be acceptable and feasible and to achieve clinically meaningful reach. The hospital sent 1526 SMS text messages signposting patients to either the NHS Smokefree or Drink Free Days apps, with 13.56% (207/1526) of the patients clicking on the embedded link to the apps. The level of reach exceeded our 5% a priori success criterion in a non–help-seeking population of patients receiving unsolicited SMS text messages, disconnected from hospital visits. The strategy was found to be acceptable both before and after implementation. The OptiMine study demonstrated the proof of concept for this novel implementation strategy, specifically signposting digital health promotion interventions at scale to at-risk individuals who are not seeking help.

A 13.56% (207/1526) click rate is promising, considering the comparable outcomes reported in the literature. For example, in a study to promote colorectal cancer screening, patients—identified from EHRs as being overdue for their colorectal screening—received an electronic message from their primary care physicians with information about their overdue status, methods to arrange a screening appointment, and a web-based risk assessment tool for colorectal cancer [33]. The intervention led to 3% of the patients requesting colorectal screening. Our 13.56% (207/1526) click rate exceeds this result. However, it is important to account for the action requested from patients, which will trigger varying levels of response, such as the 3% response rate for colorectal screenings versus the 13.56% (207/1526) click rate on our embedded link to behavioral interventions. Future research should build on this study and aim to define appropriate thresholds for e-referral interventions that correspond to different behaviors that require varying degrees of involvement from patients (eg, time and effort invested, duration of behavior, and difficulty of behavior). Furthermore, it is important to note that our SMS text messages were sent from the patients’ hospital, which would be expected to foster trust in the messages and their source and increase uptake.

Using SMS text messages to signpost patients to addictive behavior apps is a scalable implementation strategy, which can provide individualized support to patients at risk. As such, cost-effectiveness increases as the strategy is scaled up. Our low-cost and low-burden strategy has the potential to reach large number of patients at high risk in a novel form (proactive referral to existing tools). Health promotion is typically delivered in primary care and community settings, but it is equally important in acute care settings, as health behaviors such as smoking and risky drinking can cause long-term health conditions and exacerbate existing health conditions. Following the success of this study, the WSFT plans to routinely deliver health promotion advice using electronic signposting.

### Strengths and Limitations

The mixed methods design helped to explore multiple implementation outcomes that assess both proximal outcomes and indicators of success [23]. This was a novel and pragmatic study, facilitated by NHS staff in a busy hospital setting. Major strengths of this study included the real-world application of the strategy with patients at the hospital and readiness of the implementation context. Senior-level leadership and buy-in from a public health consultant within the digital health team were instrumental in the successful implementation of this project. Global Digital Exemplar Trusts have EHRs and in-house expertise to support the setup and delivery of SMS text message signposting. We identified the following stakeholders as pivotal to the successful setup, implementation, and evaluation of the strategy: patient representatives, information analysts (assess infrastructure and access to data), deputy chief information officer (design and oversight of message implementation), IT integration developer (implement SMS text message signposting and send messages), public health manager (in-house data analysis), communications team, volunteer coordinator, and administrative support. Although the readiness of the implementation context is considered a strength of our study, it could be seen as a limitation to broad scalability in less-ready, low-resourced contexts, which may serve more disadvantaged or underserved populations.

There were challenges in using the EHR as a research database. The data set was extracted directly from the EHR at different time points owing to the size of the EHR, time to download the data, and workload of hospital staff. Queries for different parts of the data were run on different days over a 4-month period from October 2019. Each day, the total number of EHR records changes owing to new records being created, existing records being edited, and people dying. Smoking and alcohol consumption status were not retrievable from hospital day patients, which meant that there were large amounts of missing data. Furthermore, there were difficulties in retrieving accurate health condition data, and matching to eligible patients was not possible. Health condition data rely on the population of the appropriate fields in a patient record; if the condition is listed in the wrong place or not recorded at all, it cannot be electronically retrieved. This occurs with some frequency across the EHR, and this limitation needs to be addressed before using electronically retrieved health data for similar studies in the future. As such, the full capacity of patient reach was not used owing to missing data, and the impact of long-term health conditions on the likelihood of clicking the embedded link is unknown.

Our primary outcome—whether each participant did or did not click the embedded link—directly measured the willingness to access information about behavior change apps among a non–help-seeking population. However, we did not measure the proportion of participants who actually downloaded and used the app. Although technical limitations prevented us from measuring app use, uptake should be assessed through future studies to support broader dissemination of this approach. In addition, response rates to the web-based questionnaire were low and may have been subject to response bias. This may have been owing to message fatigue, where patients had previously received up to 2 SMS text messages signposting to the apps, followed by 2 messages inviting participation in the survey. Finally, the SMS text messages did not include instructions for opting out of future messages, which is a requirement in the United States. Future studies should examine click rates in the presence of explicit opt-out instructions.

### Implications for Further Research and Practice

The WSFT views this approach as the future for routine delivery of digital interventions for health promotion within their acute care context and as an important adjunct to the opportunistic behavior change interventions that are made by health care professionals. Further refinement and evaluation are needed to optimize the SMS text message content and delivery approach. Additional studies with other populations are needed to understand the best strategy for implementation at other hospitals and institutions. The COVID-19 pandemic has highlighted the urgent need for scalable health promotion support, which can be delivered via digital technology. There is also an international call for greater access to high-quality, safe, and effective addictive behavior apps [34]. Regarding refinement and further evaluation of SMS text message signposting to apps, we propose the following ideas for further development:

1. Tailor message content to groups of people who are less likely to engage, such as older people, low socioeconomic status groups, and ethnic minority groups.
2. A timely trigger for the SMS text message, possibly in the context of a face-to-face consultation, may be more effective than sending all the messages at the same time. The SMS text messages were sent in January 2020 to optimize the click rate by taking advantage of the seasonal high demand for support, and therefore, other times of the year may be less or more likely to have high rates of engagement.
3. Use the strategy to target other health behaviors.
4. Explore other implementation outcomes, such as implementation cost and cost-effectiveness, compared with other methods for promoting lifestyle change; sustainability; and fidelity, including engagement with the app.
5. Investigate system interventions for addressing barriers identified regarding the reliability of EHR data.

### Conclusions

The OptiMine study demonstrated the proof of concept for this novel implementation strategy, which used SMS text messages to signpost at-risk individuals to behavior change apps at scale. The level of reach exceeded our a priori success criterion for a non–help-seeking population of patients receiving unsolicited SMS text messages, disconnected from hospital visits. These findings suggest that electronic signposting to support is an effective method for health systems to proactively engage meaningful proportions of their at-risk populations.

## Appendix

Multimedia Appendix 1 Qualitative focus group findings.

## Abbreviations

AUDIT-C: Alcohol Use Disorders Identification Test–Consumption
CONSORT: Consolidated Standards of Reporting Trials
EHR: electronic health record
NHS: National Health Service
NIHR: National Institute for Health Research
RR: relative risk
WSFT: West Suffolk NHS Foundation Trust
