# Supplementary material for: Novel Implementation Strategy to Electronically Screen and Signpost Patients to Health Behavior Apps: Mixed Methods Implementation Study (OptiMine Study)
Source: JMIR Form Res. 2022 Jul 11;6(7):e34271. doi: 10.2196/34271 (PMC9315888; doi:10.2196/34271)
Supplement: Multimedia Appendix 1 [file formative_v6i7e34271_app1.docx]

**Table S1. Qualitative focus group findings**

| **Implementation strategy component** | **Overall suggestions** | **Illustrative comments*** | **Implications for Phase 3** |
| --- | --- | --- | --- |
| **Compatibility** |  |  |  |
| Message format | »Texts were more popular, compatible with app download and hospital more likely to have patient telephone numbers »There were few email addresses in the ehr system and the patient portal was not well used and still under development » Patient choice of format was raised, but acknowledgement communication preferences were not routinely recorded » More than one format (e.g. text and email) was acceptable for some people (enhanced access and greater patient choice), but not for others (bombardment) | P021 'I can see these days that the app, sending out a text message might be a quicker, easier way of doing it'  S003 'I think a text would capture most people' | Messages to be sent as texts |
| Number of messages | »Reminders acceptable (e.g. more likely to action on another day) but not at a high frequency (would increase resistance) or too many (~3 max) »An opt out option would be ideal | P024 'it would be wonderful to have more than one message, because it does happen that I lose a message or I completely forget about it'  P020 'You don’t want this to become irritating. You just want it to flash up once or possibly twice, but three times is gonna be too much'  S004 'an opt out perhaps for people who are definitely not gonna change their mind about it' | One reminder message to be sent to patients not clicking on first app link. Limited resources to include an opt out or further reminders for this study |
| Timing of messages | »Being able to access the message at any convenient time was seen as advantageous  Staff only:  »A teachable moment e.g. after a patient visit might be impactful, however might be information overload at this time or unacceptable following e.g. a cancer diagnosis; and reaching people who do not have appointments would also be important | P017 'They can access it when they want to, not in a time dictated by something else'  S005 'It gives them the ability to do something straight away' | Messages to be sent at 12.30 and 17.00 in line with the literature |
| Message fit with routine and norms | »The messages and apps were felt to fit with patient lifestyle: ability to incorporate anonymously, to access apps whenever useful, to access support quickly, to delete messages if not wanting to change behaviour, and paper-free. However, likely to be more difficult for the older generation to access »Drinking was generally felt to be a different and more socially acceptable behaviour, unless heavily drinking »Mixed views about sending to all patients (bombardment, cost waste concerns) versus those with the behaviour (stigma, medical concerns, privacy and equality concerns, not reaching people who've not admitted to the behaviour)  Staff only:  »Fits with current culture of health promotion, patient care, and signposting to support services »Currently staff are used to seeking patient permission for referral to services. Alcohol reduction is less addressed and patients are less aware of risky drinking. Apps are not currently advised »Acknowledgement that patient data in electronic records is patchy and free text is often used (consultant culture in particular) | P023 'I like it to be speedy and I like it to be really easy'  P021 'advertising is becoming very intrusive today'  S004 'we’re now moving towards a sort of public health environment where we’re being much more proactive with patients about their care'  S002 'It would have to be targeted, so it would have to be related to a clinical issue'  S003 'I don’t think we record that often enough as structured data. So it’ll be in there as free text' | Posters and other adverts to be used to raise awareness of study for patients and staff. The patient records will be searched using a range of techniques to maximise identification of relevant patients |
| Message content | »Source coming from the hospital was important (credibility) »Positive framing was preferred. Negative messages might cause offense and were thought to be overdone e.g. on cigarette packs »Messages should be brief and attention grabbing or increase curiosity, and emphasise the apps were free »Preference for generic wording (if too detailed may cause privacy concerns and concern about being too direct) »Smoking and drinking were viewed as different behaviours and consensus not to combine behaviours in the same message »Concerns about personalising to a patient name (open to error, to cause offense, and privacy concerns) »For reminder messages, suggestion for these to come from a different angle to encourage interest  Staff only: »Mixed ways of referring to the Trust, branding was important »Mixed views on addition of a specific department or sender name e.g. 'xx chest physician' might add more impact but concerns re: clinician workload and added complication to set up | P015 'I think that’s the key, to say this is from the West Suffolk Hospital'  P022 'it’s gotta be brief'  P016 'Not everybody uses their first name. Some people prefer to be called by their first name, some people prefer to be called by their title...Is this person being a little bit too familiar?’  P024 'seeing it from a different angle in case it didn’t grab me the first time'  S014 'You want it to be positive.'  S003 'I think you’ve got to send a message either about smoking or about drinking.' | Smoking and drinking as separate message, brief content, West Suffolk Hospital as source, positive framing. Reminder message to be worded differently  Message content to be approved by West Suffolk Hospital communications, Research Ethics Committee & Health Research Authority |
| Issues or complications | Patients: **i) Legal:** litigation might be a possibility; **ii) Privacy:** feeling of 'big brother', concern of someone else picking up the message, permission for opt in but acknowledgement few might opt in, concern staff might have looked at records and remember their behaviour; **iii) Medical:** concern of inaccuracies in medical record, concern of 'am I more at risk than I thought I was?' particularly for patients with e.g. anxiety disorders, pressure may mean e.g. drinking might increase, messages might not be appropriate for those e.g. who have a cancer diagnosis; **or** **iv): Ethical or Other issues:** mixed views on whether or not to target the message to behaviour, not having the ability to contact the sender, equality and inclusivity issues e.g. for patients with disabilities, cultural issues e.g. alcohol and religion, bombardment of messages, feeling offended by message content, concern about cost or commitment if clicking the link, concern that the link may not be genuine.  Staff: **i) Legal:** messages should be approved by the hospital; **ii) Privacy:** open to concern therefore clarify messages have come from hospital i.e. data not been shared, or permission for opt in since smoking/drinking can be private behaviours; **iii) Medical:** concern of sending to 'correct' patients, some concern about validity of drinking as a problem, some concern of approaching in a 'cold' way, patients wanting to ask questions about the apps in clinics, generating additional workload, concern of appropriateness if e.g. a recent lung cancer diagnosis or recently been in detox or receiving end of life care, importance of ensuring other health promotion services are also offered to patients and ensuring the intervention is effective, possibility of further tailoring to medical condition e.g. smoking more risky for diabetics; **or iv) Ethical or Other issues:** mixed views on whether or not to target the message, bombarding patients, overload, offending patients, equality, stigma, a patient might have already expressed they want to carry on smoking/drinking (patient choice), may have to deal with patient complaints | P018 'Where have you got that information from?'  P016 'Some people may have conditions that they don’t want other people to know about'  S013 'I would imagine some people might want to know how you know I’m a smoker' | Data Protection Impact Assessment to be conducted by West Suffolk Hospital. Study contact details to be supplied in case of complaint and patients to be made aware they can contact PALS (Patient Advice and Liaison Service). Messages will not be sent to patients on end of life care, alcohol dependent patients or to patients who have opted out of communications. Messages will be worded generically |
| **Complexity** |  |  |  |
| Any difficulties for patients | »Proportion of people who won't have a smartphone/ computer skills, especially elderly (inclusivity issues). People running out of data on their phones/ unable to connect to the internet (an advantage of having a reminder message) »For those with smartphones, it was felt patients would access the apps relatively easily » Concerns of computer virus, or having to pay if click link to app »Suggestion of a link going to a supportive website with the option of having an app download to increase reach. However, also suggestion to have links straight to apps for simplicity  »Disabilities were raised as a potential issue e.g. mental illness, sight issues, manual dexterity. Suggestion to ensure the link was clear on the message.  »Agreement to ensure the process was as simple and brief as possible with few steps required | P024 'It’s got to be ultra-quick'  S001 'There’s always a cohort of people who haven’t got any data on their phone and they’ve run out of … all of that kind of thing, but by and large, most people will be fine, won’t they?'  S006 'Simplify, keep it simple, keep it basic, keep it all you’ve got to do is click on this then you’re away' | It will be clarified the link is clickable and free and that the message is from West Suffolk Hospital. Link will be direct to the One You app page for patient ease. Posters and communications will increase awareness of the messages |
| Integration into hospital EHRs | »Up to date contact details, names, and determining accurate current smoking/drinking status »Minimising complexity e.g. by not adding a personal name »The system being able to recognise whether or not patients had clicked on the link (affecting sending a reminder) »Integration into other services e.g. primary care, a future consideration »Will build on current culture in sending appointment reminders to patients  Staff only:  »Staff currently not used to this (self-referral based on patients volunteering information), would need to communicate to staff and patients »Ensuring accuracy of automated data pull, lifestyle questions not always asked and could be recorded in free text » Addressing complexities of data variables e.g. hospital encounters, those with a recent cancer diagnosis »Set-up thought to be quite manual and more labour intensive initially »Set-up with a third party text supplier and cost associated with this »Ensuring no contradictions with current patient advice »Possibility of patient complaints and added workload | P015 'So the information’s gotta be accurate to start with'  S010 'Doing a text campaign like that is new to us, yeah.'  S014 'It could generate an unpredictable workload for which we’re not prepared. I don’t think that’s all that likely, but it’s possible.' | Messages will not contain personal names. The system will be set up to recognise whether or not patients have clicked on the first link. Communications will be made to staff and patients with the research team contacts in case of issues. The database will be searched as comprehensively as possible. Costs will be monitored |
| **Relative advantage** | |  |  |
| Benefits | »Advantages were seen as outweighing disadvantages »Potentially prompting a healthier lifestyle (fewer admissions e.g. for Accident and Emergencies for alcohol, reducing repeat visits, shortening hospital stays) »Free and convenient patient access (24 hour support with no travel and quick to access, ability to access anonymously)  Patients only:  »Putting the patient in control (not having to involve health professionals)   Staff only: »Proactive, another way of getting the messages across to patients (multiple attempts), increasing patient awareness of resources and possibility of generating a feeling in patients that the health service are committed to patient wellbeing, a way of increasing awareness among drinkers of being at risk »Relatively low cost »Relatively low effort (part of lifestyle screening and automated texts once set up- currently relying on health professionals raising the subject, at a potentially difficult time) | P018 'you start trying to stop people before they get really hooked'  P024 'The benefits are putting you in control of doing something positive for your health, that’s very easy. You don’t have to ring up, you don’t have to wait and talk to somebody, you don’t have to make an appointment. You’re given the hope that you’re gonna be given some help straight away that will be in your hands'  S003 'It’s relatively low effort, relatively low cost...So these are people who will have had multiple attempts from the system to try and stop them smoking before. And if this is the one that works, brilliant  S005: 'lots of people who drink in the risky levels don’t understand the risk that they’re at...providing them with the direct link to the app gives them the opportunity to understand their risk better' | To provide justification for implementation of text-message signposting |
| Disadvantages | Patients:  »People interested can already seek support of their GP so messages might have the opposite effect, potentially seen as offensive, people wanting to be left alone »Different views re: targeting e.g. message going to the wrong person such as a non-smoker might cause offense, but also a risk people may feel 'irate to be targeted' if messages are not generic  Staff:  »A cost for initial set-up, ongoing cost for the texts, staffing implications (set up time, continual database management and unpredictable workload e.g. due to people wanting to seek more advice on smoking cessation) »A possible increase in complaints | P017 'Don’t remind me, just leave me alone, it’s my life, I’ll do with it what I want. And that is not a personal opinion'  S006 'They’re getting on my nerves at West Suffolk, they keep sending me this message.’ | Study contact details will be supplied in case of complaint and patients can contact PALS. Messages will be worded generically |
| Impact on current programmes | »Generally viewed as complementing and adding to current lifestyle programmes  Staff only:  »Potential conflict was fewer referrals to smoking services (CQUINS) or conversely increased workload for services »Comment re: ensuring apps fits with other apps offered | P015 'I think alongside whatever’s out there'  S001 'Not conflict necessarily, although I think aren’t we encouraged to refer people through that service… whether we’d be open for any criticism if we weren’t referring them' | The messages will link to the One You website which offer supportive apps for a range of behaviours |
| Other preferred services | »Desire to continue with current services alongside the new proposed messages | P017 'I think they’re good as a bolt-on, I think the more opportunities in various places that you have for encouraging people' | The messages will run alongside current health and lifestyle services |
| **Other pertinent points** |  |  |  |
| Any other pertinent points | »Interest in extending to other health behaviours »Some lack of knowledge of EHR systems »Interest in evidence base/ research outputs | S013 'We are here to try and help the public population about health as a whole. Would you like any help with …stopping smoking, reducing, having alcohol free days, losing weight, exercising, all that stuff’   Do you think you’d be interested to know how many click on the link for example? 'S006: Oh yeah, definitely.  S007: Definitely, definitely yeah' | Feedback to patients and hospital  Future research possibilities e.g. targeting versus not, extension to other health behaviours |
| * P=patient; S=staff | |  |  |
